# Mesenchymal stem cells ameliorate silica‐induced pulmonary fibrosis by inhibition of inflammation and epithelial‐mesenchymal transition

**DOI:** 10.1111/jcmm.16621

**Published:** 2021-06-02

**Authors:** Jingjing Wei, Qiuyan Zhao, Guo Yang, Ruoxuan Huang, Chao Li, Yuanmeng Qi, Changfu Hao, Wu Yao

**Affiliations:** ^1^ School of Public Health Zhengzhou University Zhengzhou China

**Keywords:** epithelial‐mesenchymal transition, mesenchymal stem cells, pulmonary fibrosis, silicosis, TGF‐β/Smad pathway

## Abstract

Silicosis is a devastating occupational disease caused by long‐term inhalation of silica particles, inducing irreversible lung damage and affecting lung function, without effective treatment. Mesenchymal stem cells (MSCs) are a heterogeneous subset of adult stem cells that exhibit excellent self‐renewal capacity, multi‐lineage differentiation potential and immunomodulatory properties. The aim of this study was to explore the effect of bone marrow‐derived mesenchymal stem cells (BMSCs) in a silica‐induced rat model of pulmonary fibrosis. The rats were treated with BMSCs on days 14, 28 and 42 after perfusion with silica. Histological examination and hydroxyproline assays showed that BMSCs alleviated silica‐induced pulmonary fibrosis in rats. Results from ELISA and qRT‐PCR indicated that BMSCs inhibited the expression of inflammatory cytokines TNF‐α, IL‐1β and IL‐6 in lung tissues and bronchoalveolar lavage fluid of rats exposed to silica particles. We also performed qRT‐PCR, Western blot and immunohistochemistry to examine epithelial‐mesenchymal transition (EMT)–related indicators and demonstrated that BMSCs up‐regulate E‐cadherin and down‐regulate vimentin and extracellular matrix (ECM) components such as fibronectin and collagen Ⅰ. Additionally, BMSCs inhibited the silica‐induced increase in TGF‐β1, p‐Smad2 and p‐Smad3 and decrease in Smad7. These results suggested that BMSCs can inhibit inflammation and reverse EMT through the inhibition of the TGF‐β/Smad signalling pathway to exhibit an anti‐fibrotic effect in the rat silicosis model. Our study provides a new and meaningful perspective for silicosis treatment strategies.

## 1 INTRODUCTION

Silicosis is a devastating occupational disease associated with long‐term silica dust particle inhalation and deposition, and it is characterized by chronic inflammatory changes, granuloma formation and diffuse pulmonary interstitial fibrosis.^1^ It is currently the most common occupational disease with the highest incidence and prevalence rates in China. Rapid industrialization has increased occupational exposure to respirable silica particles in various industries and work environments, such as coal and metal mines; building material, glass product and ceramic manufacturing; and sandblasting, as well as in iron, steel and non‐ferrous foundry work. As artificial stone has recently become widely popular in interior designing, a silicosis epidemic is emerging in the artificial stone industry.^2^ Owing to insufficient protective measures, workers form a high‐risk group for silicosis development. Silicosis may develop after many years, even with exposure cessation. Despite prevention efforts for many decades, silicosis remains a serious problem worldwide.^3^ Although the incidence of silicosis is still very high in the developing countries, many people believe, owing to rapid economic development, that silicosis is a historical and ancient disease and therefore rarely attracts clinical attention. Unlike those employed for common lung diseases such as asthma, lung cancer and other fibrotic lung diseases, modern medical research techniques are rarely used for the research and treatment of silicosis. Clinically, there are no effective treatments available for silicosis except lung transplantation.^4^, ^5^ Therefore, new therapies are desired to prevent, improve or inhibit silicosis.

Mesenchymal stem cells (MSCs) are adult multipotent progenitor stem cells with self‐renewal capacity and multi‐lineage differentiation potential.^6^ MSCs have low immunogenicity, multi‐differentiation potential, immunomodulatory properties, paracrine function, homing effect and abundant sources and are easily isolated and amenable to culture expansion in vitro. Thus, MSCs are considered promising candidates for clinical applications, such as neurodegenerative diseases,^7^ cardiovascular diseases^8^ and lung diseases.^9^, ^10^ Till date, multiple studies have focused on MSCs.

Silicosis initiation and development are a complex process involving multiple cells regulated by complex molecular networks. Silicosis begins with inflammation, alveolar epithelial cell damage and apoptosis, followed by tissue repair, epithelial‐mesenchymal transition (EMT) stimulation, myofibroblast activation, fibroblast and myofibroblast foci formation, and extracellular matrix (ECM) deposition, ultimately leading to pulmonary fibrosis. Intratracheal Au nanoparticle–labelled or unlabelled bone marrow–derived mesenchymal stem cells (BMSCs) transplantation into mice with silica‐induced pulmonary fibrosis has shown that BMSCs inhibit silica‐induced macrophage activation and have an anti‐inflammatory and anti‐fibrotic role.^11^ During EMT, epithelial cells transdifferentiate into mesenchymal cells, which is essential for embryogenesis and tissue regeneration. EMT also contributes to fibrotic disease^12^ and cancer progression.^13^ During this process, loss of cell‐cell adherens junctions and apical‐basal polarity triggers cytoskeletal composition shift, and cell‐cell and cell‐ECM interactions are remodelled.^14^ Epithelial cells lose their polarity and acquire mesenchymal phenotypes such as high migration and invasion, anti‐apoptosis and ECM‐degrading ability. EMT involves the functional cooperation of several signalling pathways, such as transforming growth factor β (TGF‐β), Notch, Wnt/β‐­catenin and Hedgehog. Among these, TGF‐β family signalling plays a predominant role.^15^ The Smad protein family is the downstream substrate of the TGF‐β receptor kinase present in the cytoplasm. It directly transduces the TGF‐β signals from the cell membrane to the nucleus and is an important signalling molecule in the TGF‐β signalling pathway. A study has reported that BMSCs and their exosomes promote endometrial repair and reverse EMT via the TGF‐β1/Smad signalling pathway.^16^ Another study has shown that adipose‐derived stem cell (ADSC) transplantation attenuates renal interstitial fibrosis through inhibition of EMT via the TGF‐β1 signalling pathway.^17^

Therefore, we investigated the effects of BMSCs on silica‐induced pulmonary fibrosis in rats. The underlying mechanism was also discussed. This study provides a theoretical basis for silicosis treatment.

## 2 MATERIALS AND METHODS

### 2.1 Culture and identification of BMSCs

Sprague‐Dawley (SD) rat BMSCs were purchased from Cyagen Biosciences Inc (Suzhou, China). The cells were recovered and seeded into T25 culture flasks with sufficient complete medium (Cyagen) in a saturated humidified atmosphere with 5% CO_2_ at 37°C. The medium was exchanged every 3 days, and when the cells reached 80%‐90% confluence, they were passaged. BMSC morphology was observed under a light microscope. The multi‐lineage differentiation of BMSCs was determined using adipogenic and osteogenic differentiation medium (Cyagen). The passage 2 cells (2 × 10^4^ cells/cm^2^) were seeded into two six‐well plates and cultured with the corresponding differentiation medium, and Oil Red O staining and Alizarin Red staining were performed, respectively, after 4 weeks and observed under a light microscope. The passage 3 cells were harvested, washed and resuspended in PBS, and incubated with anti‐CD90‐FITC, anti‐CD29‐PerCP‐eFluor 710, anti‐CD45‐APC and anti‐CD11b‐PE (eBioscience, San Diego, CA, USA) or isotype control antibodies (eBioscience) for 30 minutes at 4°C in dark. After incubation, the cells were washed and analysed using an Accuri C6™ Plus Flow Cytometer (BD Bioscience).

### 2.2 Animals

All animals received humane care, and all methods were carried out in accordance with the Guide for the Care and Use of Laboratory Animals. The experiments were approved by the Committee on the Ethics of Animal Experiments of Zhengzhou University. Six‐week‐old male SD rats weighing about 200 g were purchased from Beijing Vital River Laboratory Animal Technology Co., Ltd. (Beijing, China) and maintained in the specific pathogenfree (SPF)–grade animal room in College of Public Health, Zhengzhou University.

### 2.3 Rat model of silicosis and BMSC treatment

After one week of adaptive breeding, these animals were randomly divided into three groups: control group, silica group and BMSCs group (n = 20 rats per group, total 60 rats). Crystalline silica particles (0.5‐10 μm, approximately 80% between 1 and 5 μm; Sigma‐Aldrich, USA) were autoclaved and suspended in sterile saline at a concentration of 100 mg/mL. Silica suspensions were sonicated for 10 minutes before use. After the animals were anaesthetized by isoflurane inhalation, the silica and BMSCs group rats received non‐exposed intratracheal instillation of 500 μL silica suspension (100 mg/mL/rat). The rats in the control group were treated with 500 μL sterile saline. Then, 1 mL BMSCs (2 × 10^6^ cells/mL) were injected into rats of the BMSCs group by tail vein, whereas an equal volume of sterile saline was injected in the control and silica group rats on days 14, 28 and 42 after silica suspension administration. A total of 10 rats in each group were killed on days 28 and day 56 after intratracheal instillation. After intraperitoneal anaesthesia with pentobarbital sodium (2%, 50 mg/kg), the rats were weighed and fixed on an anatomic device. After collecting the bronchoalveolar lavage fluid (BALF), the complete lung tissue was removed, the trachea and fascia were obtuse removed, washed with PBS and dried on a filter paper. The lung was accurately weighed, and the lung coefficient was calculated as the ratio of lung weight (g) to bodyweight (g) × 100. Lung tissue and BALF were stored for subsequent analyses. The study design is shown in Figure S1.

### 2.4 Histological examination

The left lungs were fixed with 4% paraformaldehyde, embedded in paraffin and then cut into 6‐μm‐thick paraffin sections. The sections were, respectively, stained with haematoxylin and eosin (H&E) to observe lung morphology and inflammatory infiltrates and Masson staining to estimate fibrosis severity. A semi‐quantitative score of pulmonary inflammation and fibrosis was performed according to the inflammation score^18^ and modified Ashcroft scale.^19^ For immunohistochemical (IHC) staining, sections were incubated with rabbit anti‐rat primary antibodies against fibronectin (1:600), collagen Ⅰ (1:1000), E‐cadherin (1:1000) and vimentin (1:5000) (both from ProteinTech, Rosemont, IL, USA), followed by a horseradish peroxidase (HRP)–conjugated goat anti‐rabbit IgG secondary antibody (1:5000, ProteinTech). The whole section was then scanned, and the images were obtained using CaseViewer.

### 2.5 Hydroxyproline assay

To analyse pulmonary fibrosis severity, pulmonary hydroxyproline (HYP) content was measured using hydroxyproline detection kit according to the manufacturer's instructions (Nanjing Jian Cheng Institute, Nanjing, China). The HYP levels in lung tissue were detected at an absorbance of 550 nm. The results were expressed as μg HYP/mg of lung weight.

### 2.6 Enzyme‐linked immunosorbent assay (ELISA)

The lung tissues were homogenized using saline. The homogenates and collected BALF were centrifuged for ELISA. TNF‐α, IL‐1β, IL‐6 and TGF‐β1 levels in the lung tissues and BALF were measured using ELISA detection kits according to the manufacturer's instructions (Elabscience, Houston, TX, USA).

### 2.7 Quantitative reverse transcription polymerase chain reaction (qRT‐PCR)

The lung tissues were homogenized using saline. Total RNA was extracted from the homogenates using RNAiso Plus Reagent (Takara, Kyoto, Japan) and quantified using a NanoDrop 2000 Spectrophotometer (Thermo Fisher Scientific). Total RNA was reverse‐transcribed into cDNA using the PrimeScript™ RT Reagent Kit with gDNA Eraser (Takara). qRT‐PCR was performed with SYBR Green PCR Kit (Takara) and an ABI 7500 Fast Real‐Time PCR System (Applied Biosystems). All samples were assayed in triplicates. All results were calculated using the 2^−ΔΔCt^ method and normalized to that of GAPDH. The primer sequences are listed in Table S1.

### 2.8 Western blot

The lung tissues were lysed using radioimmunoprecipitation assay (RIPA) lysis buffer containing phenylmethanesulphonyl fluoride (PMSF), and the protein concentration was measured using a bicinchoninic acid (BCA) assay kit (Boster, Wuhan, China). The proteins were electrophoresed on sodium dodecyl sulphate‐polyacrylamide gels and transferred to polyvinylidene fluoride (PVDF) membranes (Millipore, USA). After blocking with 5% milk for 2 h on a shaker, the membranes were incubated overnight at 4°C with rabbit anti‐E‐cadherin (1:1000, Servicebio, China), rabbit anti‐Vimentin (1:1000, Servicebio), rabbit anti‐Collagen I (1:1000, Abcam, USA), rabbit anti‐Fibronectin (1:5000, ProteinTech), rabbit anti‐TGF‐β1 (1:1000, Abbkine, China), mouse anti‐Smad2 (1:5000, ProteinTech), rabbit anti‐phosphorylated‐Smad2 (1:5000, Abcam), rabbit anti‐Smad3 (1:500; Affinity Biosciences, USA), rabbit anti‐phosphorylated‐Smad3 (1:1000, Affinity), rabbit anti‐Smad7 (1:500; Affinity Biosciences) and mouse anti‐GAPDH (1:20 000, ProteinTech). Next, the membranes were washed with Tris‐buffered saline/Tween (TBST) thrice and incubated with HRP‐conjugated secondary antibodies (1:20 000, ProteinTech) for 1 hour at room temperature. Protein bands were detected using an enhanced chemiluminescence (ECL) kit (Absin, Shanghai, China) and imaged on Amersham Imager 600 (Amersham, Little Chalfont, UK). The intensities were analysed and quantified using ImageJ software to calculate relative expression by normalizing to GAPDH.

### 2.9 Statistical analysis

All data obtained from the experiments were presented as mean ±standard deviation (SD). Comparisons between two groups were analysed using the Student's *t* test. Comparisons among multiple groups were performed with one‐way analysis of variance (ANOVA). Statistical analysis was performed with SPSS 21.0. Values of *P* < .05 were considered statistically significant.

## 3 RESULTS

### 3.1 Identification of BMSCs

**FIGURE 1:**
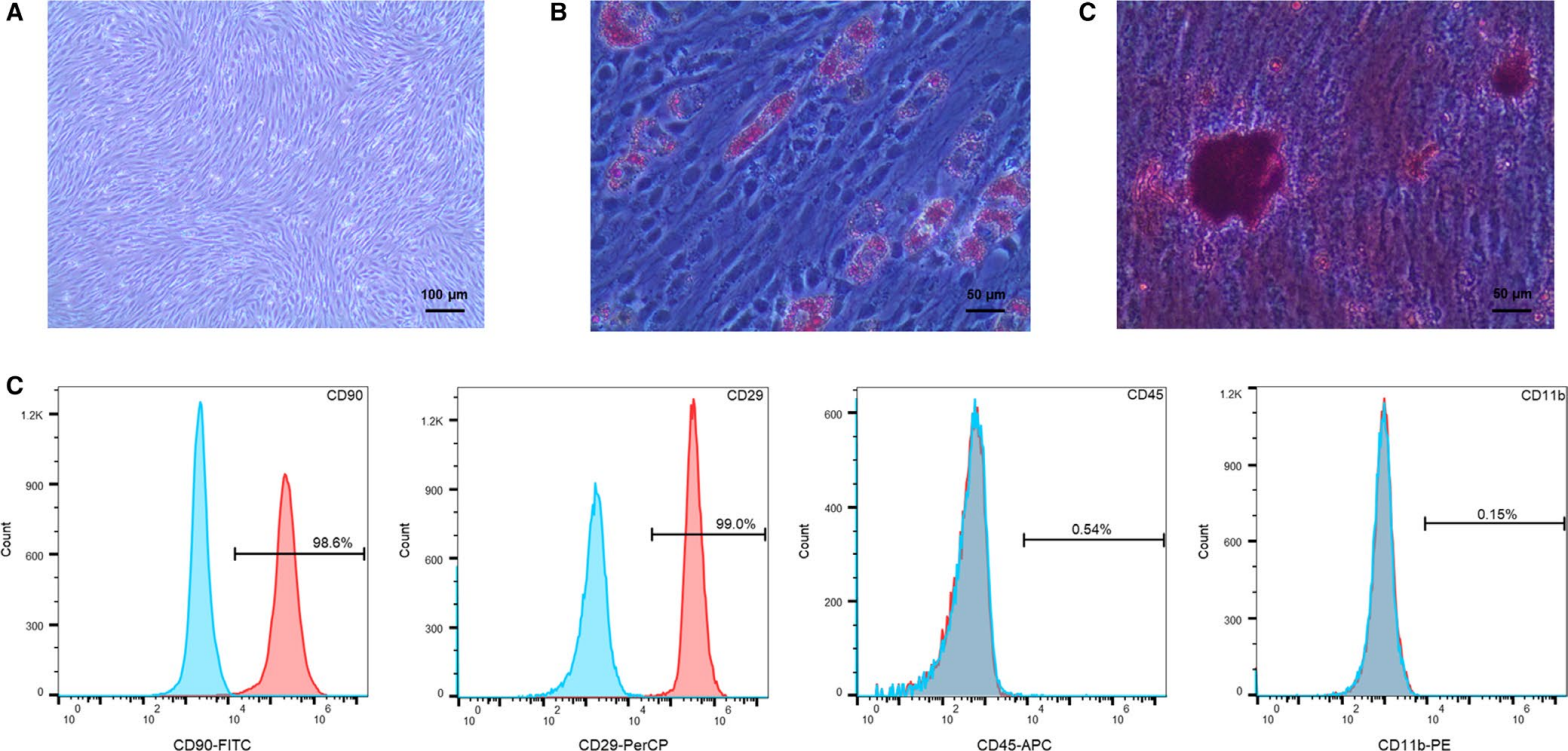
Characterization of BMSCs. (A) The morphology of passage 3 BMSCs was observed by an optical microscope (100×). (B) Adipogenic induction of BMSCs was shown via Oil Red staining (200×). (C) Osteogenic induction of BMSCs was shown via Alizarin Red staining (200×). (D) Detection of CD90, CD29, CD45 and CD11b by flow cytometry

We confirmed the biological characteristics and purity of BMSCs by observing the cell morphology under a light microscope, determining the differentiation ability from adipocyte and osteogenic induction, and detecting the cell surface markers using flow cytometry. The cells were spindle‐shaped or fibroblast‐like, converged and arranged in a swirl (Figure 1A). Lipid droplets and red calcium nodules of various sizes were visualized by Oil Red O (Figure 1B) and Alizarin Red (Figure 1C) staining, respectively, which demonstrated that the cells exhibited the capacity to differentiate into adipocytes and osteoblasts under the induction of adipogenic and osteogenic differentiation medium, respectively. In addition, flow cytometry analysis showed that CD90 and CD29 expression was 98.6% and 99.0%, respectively, whereas CD45 and CD11b expression was <1% (Figure 1D).

### 3.2 BMSCs improved lung tissue morphology of and lung coefficient

The altered lung morphology of rats in each group was observed by the naked eye. The lungs of the control group rats were red, uniformly coloured, soft and elastic, and the surface of the pulmonary lobes was smooth without spots or nodules. On the contrary, the silica group rats showed visibly injured lungs; their lungs were grey, hard and lacked elasticity, with different‐sized spots and nodules in the pulmonary lobes. Future, the lung volume of the silica group rats increased compared with that of the control group rats. Although scattered spots and nodules were present in the lungs of the BMSCs group rats, lung injury was lesser than that in the silica group rats (Figure S2). Lung coefficient is another indicator of lung injury. As shown in Table 1, compared with that of the control group, the lung coefficient of the silica group rats was significantly increased both on days 28 and 56, BMSC transplantation reduced the increase in lung coefficient caused by silica‐induced pulmonary fibrosis on day 56, but no statistical difference was observed on day 28.

**TABLE 1:** Lung coefficient of rats after surgery

Groups	28 days	56 days
Control group	0.37 ± 0.03	0.27 ± 0.04
Silica group	0.69 ± 0.10^***^	0.95 ± 0.12^***^
BMSCs group	0.64 ± 0.04^ns^	0.83 ± 0.04^#^

Note

Lung coefficient of each group. Data were presented as mean ±SD. n = 7 rats per group, ^***^ *P* <.001 vs control group. ^#^ *P* <.05, ns, non‐significant vs silica group.

### 3.3 BMSCs ameliorated the pulmonary pathological changes and pulmonary fibrosis induced by silica

**FIGURE 2:**
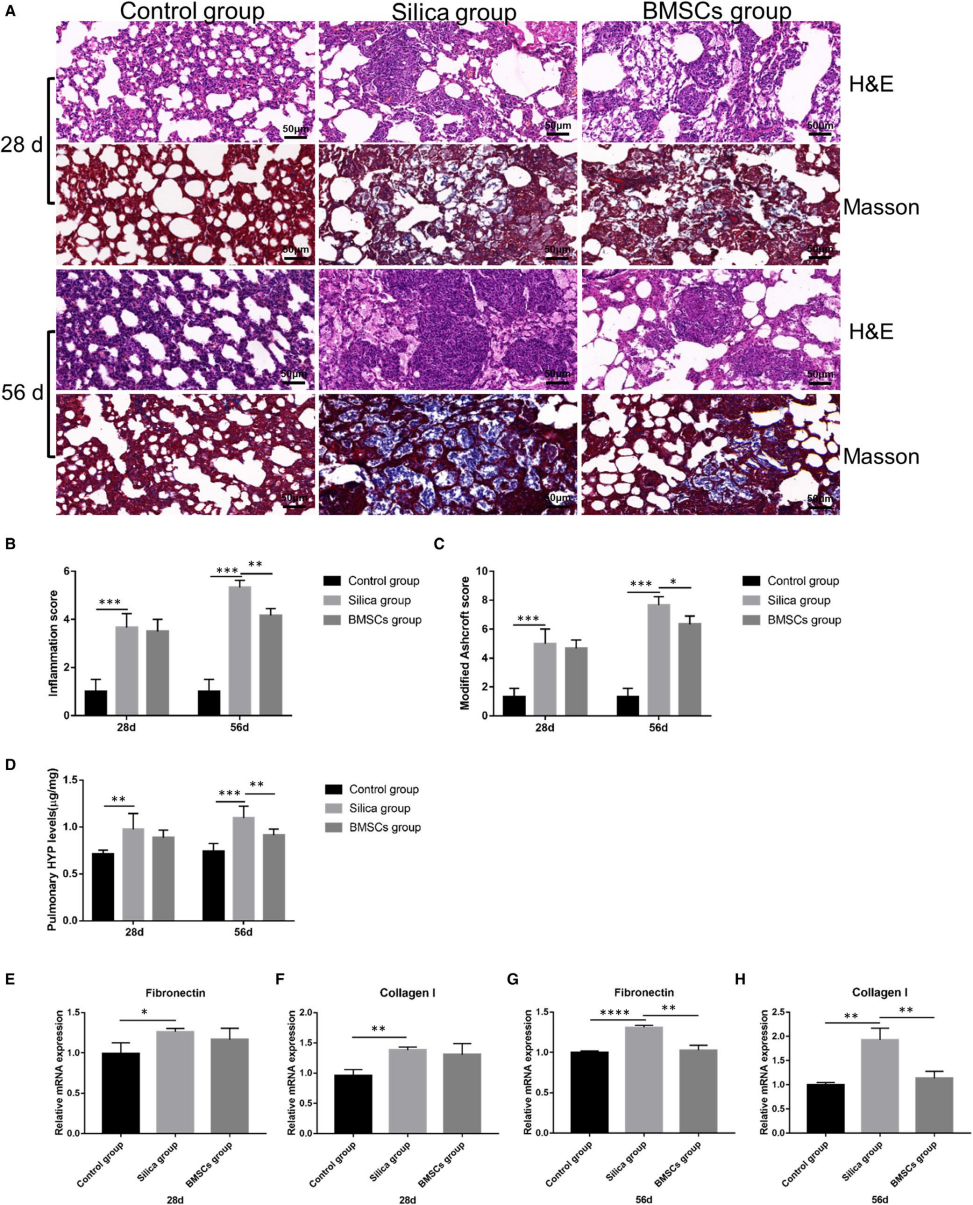
BMSCs improved pulmonary pathological changes and pulmonary fibrosis. (A) H&E staining and Masson's Trichrome Staining were performed to detect the pathological changes in lung tissues after rats were exposed to silica for 28 and 56 days (200×). (B‐C) Severity of pulmonary inflammation and fibrosis evaluated by inflammation score and modified Ashcroft score. n = 7 rats per group. (D) Pulmonary HYP content of each group after rats was exposed to silica for 28 and 56 days. (E‐H) The mRNA expression of fibronectin and collagen Ⅰ of each group after rats were exposed to silica for 28 and 56 days. n = 3 rats per group. mRNA expression values were normalized to GAPDH. Data were presented as mean ±SD. ^*^ *P* <.05, ^**^ *P* <.01, ^***^ *P* <.001, ^****^ *P* <.0001

To detect the pathological changes, the lung tissues were stained with H&E and Masson to observe inflammatory infiltrates and fibrosis, respectively. The alveolar structures in the control group were intact and distinct. In contrast, the alveolar structure in the silica group was significantly destroyed, accompanied by a large area of inflammatory cell infiltration and collagen fibre deposition both on days 28 and 56. After BMSCs transplantation, alveolar damage, inflammatory cell aggregation and collagen fibre deposition were significantly reduced (Figure 2A‐C, Figure S3). HYP content reflects collagen metabolism in tissues and is an index of disease fibrosis. HYP content in lung tissue of the silica group was significantly increased compared with that in the control group, whereas compared with the silica group, HYP content in lung tissue of the BMSCs group was significantly decreased on day 56, but no statistical difference was observed on day 28 (Figure 2C). The mRNA expression levels of fibronectin and collagen Ⅰ detected by qRT‐PCR showed the same trend (Figure 2D‐H).

### 3.4 BMSCs alleviated silica‐induced inflammatory cytokine production

**FIGURE 3:**
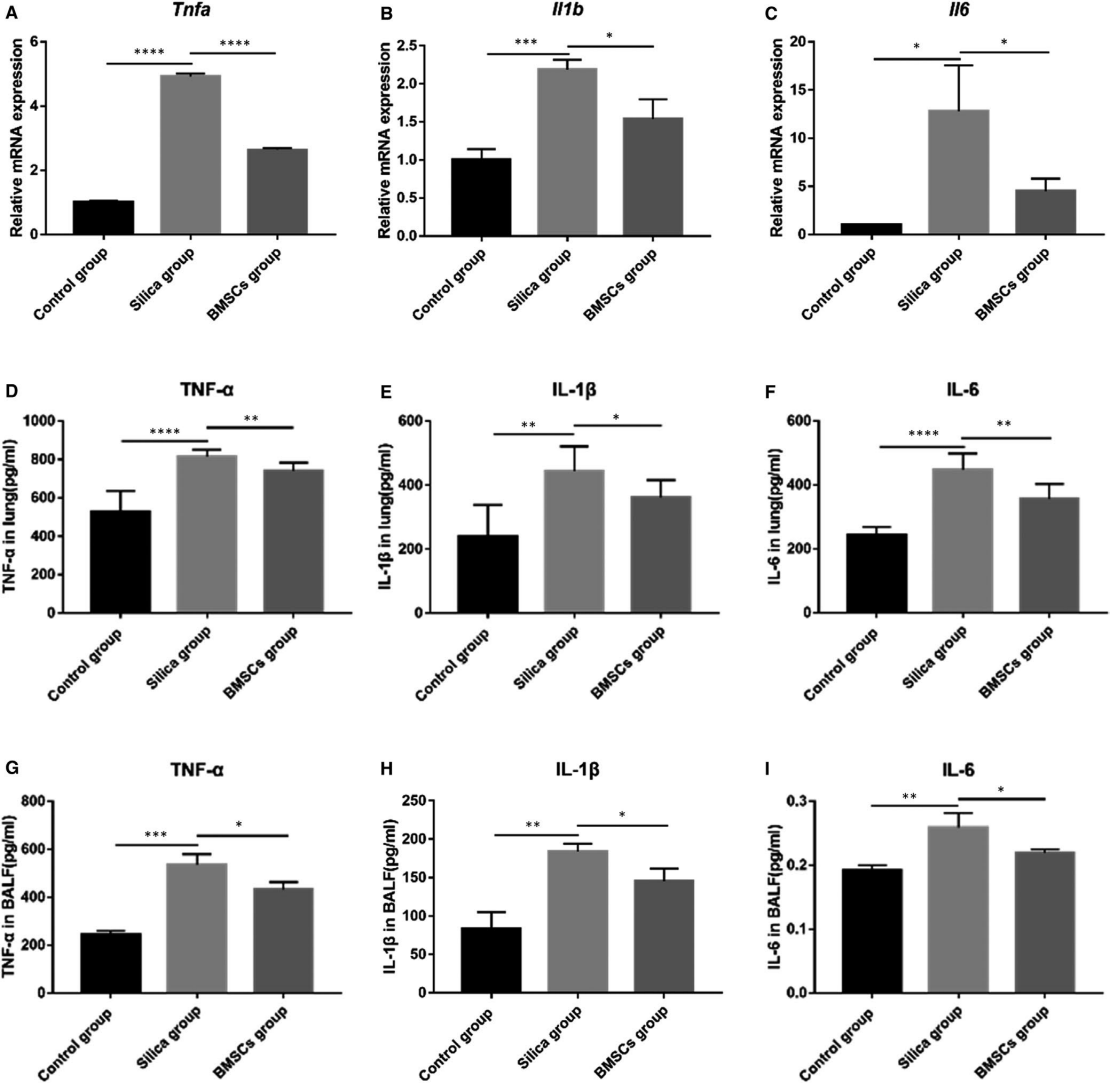
BMSCs inhibited silica‐induced inflammatory cytokine production. (A‐C) The mRNA expression of inflammatory cytokines *Tnfa*, *Il1b* and *Il6* in lung tissue. n = 3 rats per group. (D‐F) The protein expression levels of TNF‐α, IL‐1β and IL‐6 in lung tissues determined by ELISA. n = 7 rats per group. (G‐I) The protein expression levels of TNF‐α, IL‐1β and IL‐6 in BALF determined by ELISA. n = 3 rats per group. mRNA expression values were normalized to GAPDH. Data were presented as mean ± SD. ^*^ *P* <.05, ^**^ *P* <.01, ^***^ *P* <.001, ^****^ *P* <.0001

The expression levels of inflammatory cytokines including TNF‐α, IL‐1β and IL‐6 in each group were examined by qRT‐PCR and ELISA. The qRT‐PCR results showed that compared with the control group rats, the mRNA levels of *Tnfa*, *Il1b* and *Il6* in lung tissues of the silica group were significantly increased. However, compared with those in the lung tissues of the silica group rats, the mRNA levels of *Tnfa*, *Il1b* and *Il6* in the lung tissues of the BMSCs group were significantly decreased on day 56 (Figure 3A‐C). Similarly, ELISA indicated that TNF‐α, IL‐1β and IL‐6 secretion in lung tissue (Figure 3D‐F) and BALF (Figure 3G‐I) was significantly elevated in the silica group, whereas BMSCs inhibited the silica‐induced TNF‐α, IL‐1β and IL‐6 secretion on day 56.

### 3.5 BMSCs reserved silica‐induced EMT

**FIGURE 4:**
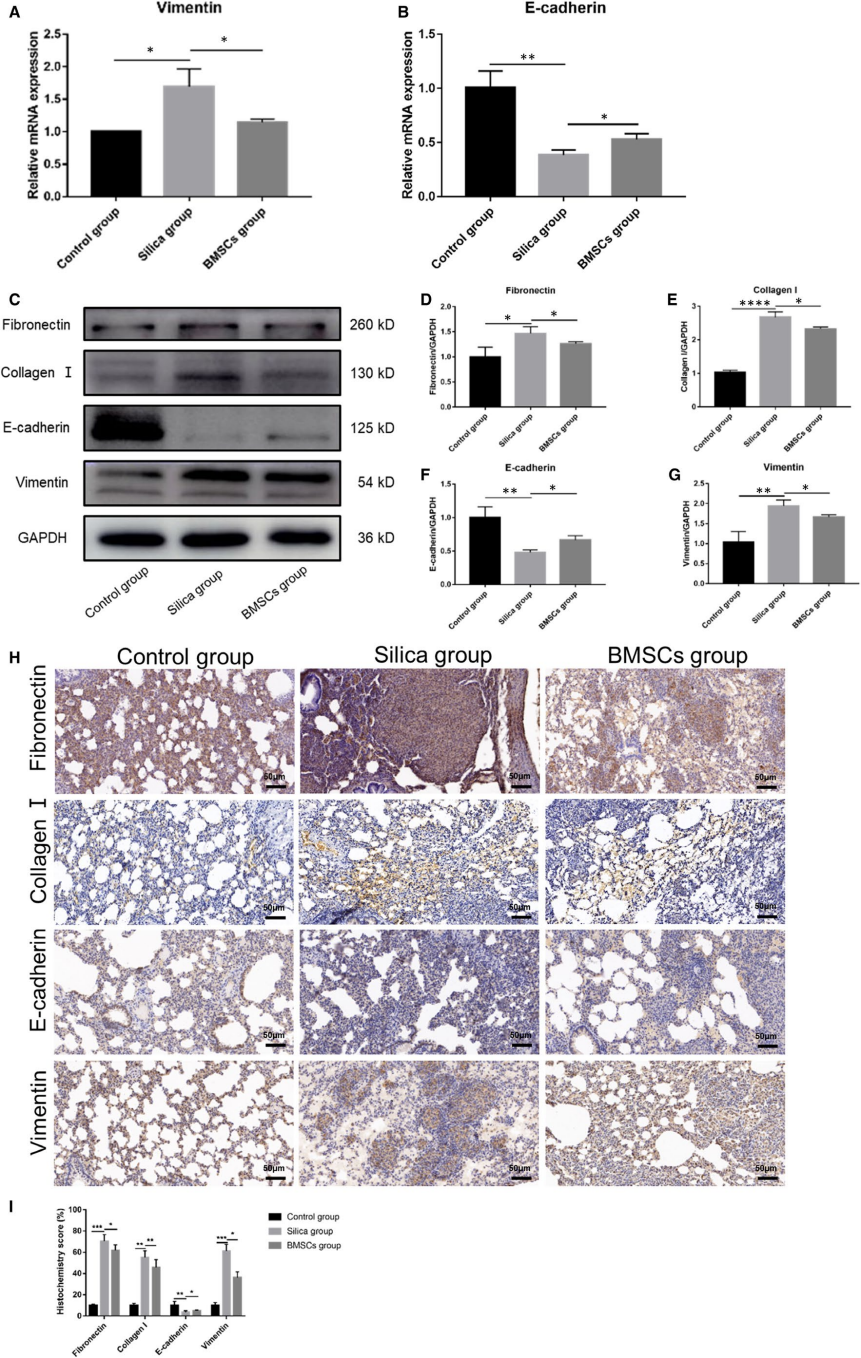
BMSCs inhibited silica‐induced epithelial‐mesenchymal transition (EMT). (A‐B) The mRNA expression of EMT‐associated markers E‐cadherin and vimentin. n = 3 rats per group. (C) Western blot results of fibronectin, collagen Ⅰ, E‐cadherin and vimentin. (D‐G) The quantitative protein expression of fibronectin, collagen Ⅰ, E‐cadherin and vimentin. n = 3 rats per group. (H) IHC staining for fibronectin, collagen Ⅰ, E‐cadherin and vimentin (200×). (I) Quantification of IHC staining, 10 random field measurements from 7 rats per group. mRNA expression values were normalized to GAPDH. Data were presented as mean ± SD. ^*^ *P* <.05, ^**^ *P* <.01, ^***^ *P* <.001

In addition to the anti‐inflammatory effects, we also explored another biological role of BMSCs in reducing silicosis. We performed qRT‐PCR, Western blot and immunohistochemistry to measure the expression levels of several EMT indicators and ECM components such as fibronectin and collagen Ⅰ. As shown in Figure 4A‐C, F‐G, the expression of epithelial marker E‐cadherin decreased significantly, and the mesenchymal marker vimentin increased in the silica group rats. However, after three BMSCs treatments, E‐cadherin expression increased and vimentin expression decreased. At the same time, we also tested the expression of ECM components fibronectin and collagen Ⅰ. Under the stimulation of silica for 56 days, ECM deposition was significant; however, BMSCs reduced the ECM deposition (Figure 2E‐F, Figure 4C‐E, H‐I).

### 3.6 BMSCs blocked TGF‐β/Smad pathway activation

**FIGURE 5:**
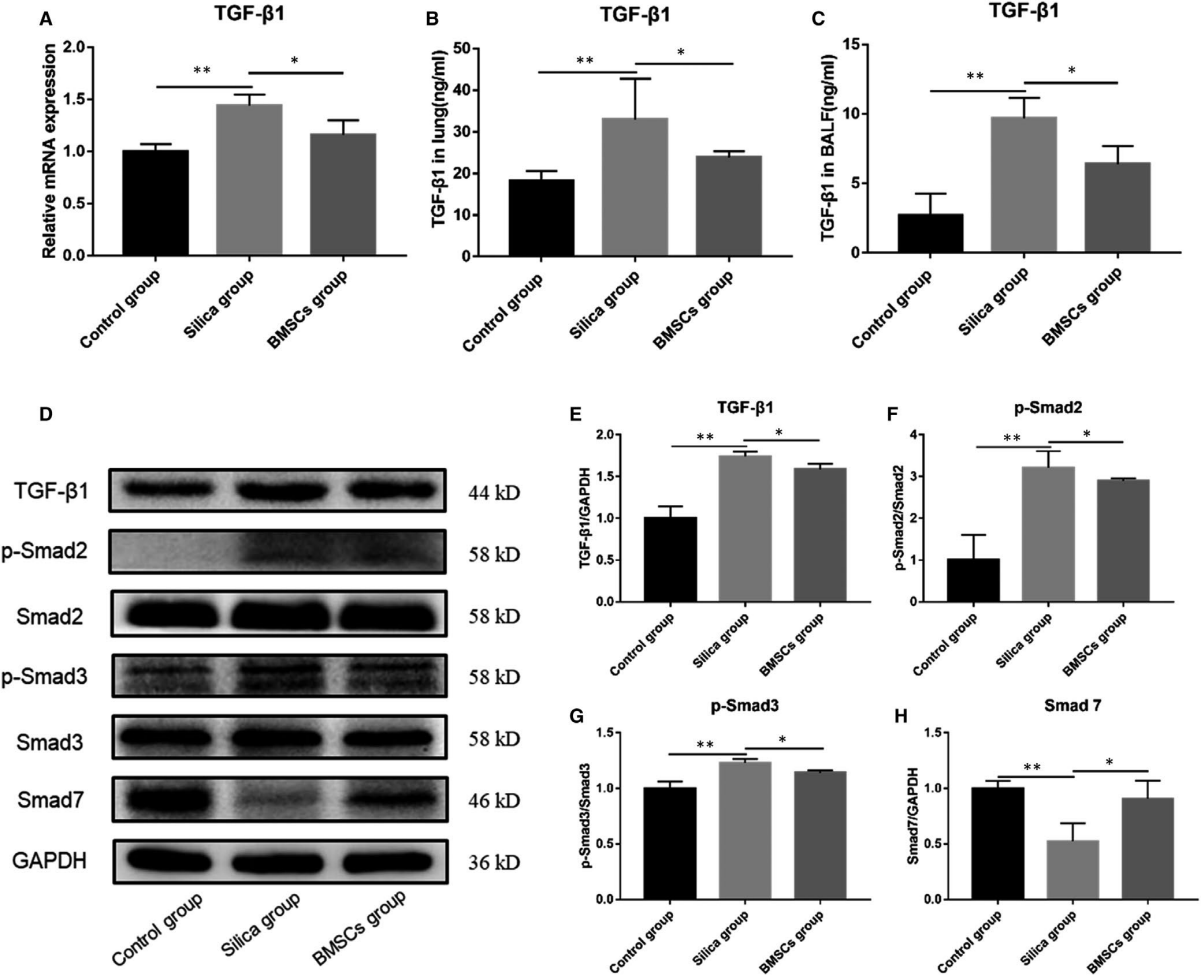
BMSCs blocked the activation of TGF‐β/Smad pathway. (A) The mRNA expression of TGF‐β1 in lung tissue. n = 3 rats per group. (B) The protein expression levels of TGF‐β1 in lung tissues detected by ELISA. n = 7 rats per group. (C) The protein expression levels of TGF‐β1 in BALF detected by ELISA. n = 3 rats per group. (D) Western blot results of TGF‐β1, Smad2, p‐Smad2, Smad3, p‐Smad3 and Smad7 protein expression levels. n = 3 rats per group. (E‐H) The quantitative protein expression of TGF‐β1, p‐Smad2, p‐Smad3 and Smad7 in lung tissues. mRNA expression values were normalized to GAPDH. Data were presented as mean ± SD. ^*^ *P* <.05, ^**^ *P* <.01

We further explored the mechanism by which BMSCs could alleviate EMT. qRT‐PCR, Western blot and ELISA were performed to measure the mRNA and protein expression levels of TGF‐β1. These results showed the TGF‐β1 was significantly up‐regulated in the silica group rats, whereas the mRNA and protein expression levels of TGF‐β1 were significantly decreased in the BMSCs group rats compared with those in the silica group rats (Figure 5A‐E). Western blot analysis showed p‐Smad2/Smad2 and p‐Smad3/Smad3 protein levels increased and Smad7 protein levels decreased in the silica group rats compared with those in the control group rats, demonstrating that silica activates the TGF‐β/Smad pathway. However, after BMSCs treatment, expressions of p‐Smad2/Smad2 and p‐Smad3/Smad3 were down‐regulated and that of Smad7 was up‐regulated compared with that in the silica group rats (*P* < .05) (Figure 5D, F‐H).

## 4 DISCUSSION

Stem cell–based therapies have generated substantial interest as potential approaches for a wide variety of diseases. Numerous pre‐clinical studies have been conducted on the application of MSCs to silicosis treatment,^20^, ^21^, ^22^, ^23^ but few clinical studies have been on cell‐based therapies for silicosis. In the past, most animal experiments transplanted MSCs immediately after constructing the silicosis model using silica. However, in real life, most people exposed to dust and may suffer from silicosis are workers with poor economic conditions. It is unrealistic to perform stem cell therapy before or shortly after dust exposure. They often receive treatment after symptoms or even a diagnosis of silicosis. The pathogenesis of silicosis generally includes acute inflammation, chronic inflammation and fibrosis; however, the boundaries between the stages are not clear. Our previous studies have shown that inflammation is predominant before 14 days, and fibrosis is predominant afterwards in a silica‐induced mouse pulmonary fibrosis model. After 28 days, mature cellular silicon nodules are formed, accompanied by a large amount of collagen deposition.^24^, ^25^ A recent study has reported the collagen fibres and the number of nodules formed on day 15 after silica instillation.^26^ In summary, we transplanted BMSCs on 14 days after perfusion of the silica suspension. It has been reported that BMSCs begin to decline gradually and disappear within 30 days in the body.^27^ To maintain a high level of BMSCs in the silica‐exposed rats, we repeated the intervention three times every 14 days, and rats were killed after 28 and 56 days after a single and three interventions, respectively. We also calculated the lung coefficients and detected their lung HYP content and fibrosis‐associated genes on days 28 and 56. These results showed that BMSCs reduced lung injury and improved pulmonary fibrosis on day 56, but the effect was not significant on day 28, suggesting that continuous BMSCs transplantation ameliorated silicosis in rats.

After proving that three BMSCs transplantations may be used to treat silica‐induced pulmonary fibrosis in rats, we investigated the mechanism through which BMSCs protect against silicosis in rats. Therefore, in the follow‐up experiments we only tested the rats on day 56. Silicosis is a typical pneumoconiosis associated with the inhalation of crystalline silica particles, which triggers inflammatory reactions and abnormal tissue repair in the pulmonary parenchyma, inducing irreversible lung damage and lung dysfunction. Inflammation plays a key role in the process of most interstitial diseases, if chronic and ongoing, to fibrosis.^28^, ^29^ Numerous studies have shown that MSCs or their paracrine factors are anti‐inflammatory and alleviate organ fibrosis by reducing inflammation.^30^, ^31^ To explore whether BMSCs have anti‐inflammatory effects on silica‐induced pulmonary fibrosis in rats, we measured the mRNA and protein expression levels of inflammatory factors in rat lung tissues. The results showed that inflammatory factors TNF‐α, IL‐1β and IL‐6 expression were significantly up‐regulated by silica, which indicated that the rats still had significant inflammatory response 56 days after exposure to silica suspension, but after BMSCs treatment, both mRNA and protein levels of inflammatory factors were down‐regulated, suggesting that BMSCs had anti‐inflammatory effects in silica‐induced pulmonary fibrosis in rats.

In addition, we explored other processes involved in BMSCs‐induced silica‐induced pulmonary fibrosis amelioration. EMT describes the process by which epithelial cells lose their functionality and characteristics and adopt mesenchymal characteristics that confer motility. There is increasing evidence that sustained EMT is the key mechanism of multi‐organ fibrosis pathology.^12^ E‐cadherin, the most important epithelial cells marker, is responsible for maintaining cell adhesion. The mesenchymal phenotype is apparent from the expression of mesenchymal cytoskeletal proteins, including vimentin, and the increased deposition of ECM components, such as collagen Ⅰ and fibronectin.^32^ We found that compared with the control group, E‐cadherin was significantly down‐regulated in the silica group rats, whereas the mesenchymal marker vimentin was up‐regulated; moreover, the mRNA and protein expression of collagen Ⅰ and fibronectin was also significantly up‐regulated, suggesting that silica activated the EMT in rat lung tissue, leading to the ECM deposition. After BMSCs transplantation, E‐cadherin was up‐regulated, whereas vimentin, collagen Ⅰ and fibronectin were down‐regulated. As silica particles are not easily removed from the body, pulmonary epithelial cells respond to persistent inflammation and sustained EMT, leading to silicosis. However, BMSCs reduced the inflammatory response and inhibited EMT, thus improving silicosis in rats.

We initially explored the protective effect of BMSCs against silica‐induced pulmonary fibrosis in rats through anti‐inflammatory effects and EMT reversal, and further explored the possible mechanisms. EMT signalling is a complex non‐linear network of various signalling pathways that converge or cooperate with other pathways.^33^ The TGF‐β/Smad pathway is one of the most important signalling pathways. Numerous studies have suggested that MSCs ameliorate organ fibrosis, including bleomycin‐induced pulmonary fibrosis,^34^ by inhibiting TGF‐β/Smad pathway.^35^, ^36^, ^37^, ^38^ However, previous studies have not focused on silica‐induced pulmonary fibrosis. Thus, whether BMSCs also regulate EMT through the TGF‐β/Smad pathway in silica‐induced pulmonary fibrosis is unknown. TGF‐β1 is a secreted cytokine that regulates cell proliferation, cell differentiation, apoptosis and matrix accumulation and promotes EMT. Normally, it mainly exists in the ECM in an inactive state. In this study, we found that TGF‐β1 was up‐regulated after exposure to silica particles, but decreased after BMSC transplantation. In the canonical Smad pathway, TGF‐β cell surface receptors (TβRI) recruit and phosphorylate Smad2 or Smad3, which forms heteromeric complexes with Smad4. These transcription factor complexes then translocate into the nucleus and cooperate with other transcription regulators to regulate target gene expression.^39^ Conversely, Smad7 plays a negative regulatory role and suppresses TGF‐β–induced EMT.^32^ Consistent with TGF‐β1 expression levels, phosphorylated Smad2 and phosphorylated Smad3 expression levels were decreased in the BMSC group rats, whereas negative regulatory protein Smad7 expression level was increased. Silica stimulation activated the TGF‐β/Smad signalling pathway, whereas BMSCs inhibited this activation. Although this study did not clarify the specific mechanism of BMSC‐mediated silica‐induced pulmonary fibrosis amelioration in rats, the findings contribute to the understanding of main mechanisms of silicosis. Simultaneously, we proved that the TGF‐β/Smad pathway is involved in BMSCs‐induced silicosis amelioration, but further studies are needed to reveal the specific molecular mechanisms.

Currently, there are no specific drugs and strategies for treating silicosis, and the main treatment methods include drugs such as pirfenidone and nintedanib, corticoid short‐course treatment, bronchoalveolar lavage and symptomatic treatment. Patients with end‐stage silicosis can undergo lung transplantation, but it is expensive. More than 800 clinical trials have established safety studies of MSCs.^40^ Some researchers have compared the therapeutic effects of MSCs and nintedanib on experimental pulmonary fibrosis in rats and found that their therapeutic effects were similar.^34^ These findings can push future stem cell transplantation studies in a new direction. Corticosteroids are extensively used to treat asthma and chronic obstructive pulmonary disease because of their strong anti‐inflammatory ability, which is attributed to the repression of target pro‐inflammatory gene transcription through inhibiting nuclear factor‐κB (NF‐κB) and activator protein‐1 (AP‐1) activation.^41^ Previous clinical studies have reported acute silicosis and chronic silicosis respond to corticosteroids therapy.^42^, ^43^ A recent study has also reported therapeutic treatment with intranasal glucocorticoid flunisolide improves inflammation and lung function in silica‐challenged mice. However, whether it affects the process of fibrosis remains controversial.^44^ Despite the long history of corticosteroid administration, their therapeutic effectiveness and safety remain controversial. Long‐term corticosteroid use is associated with an increased risk of adverse events, including osteoporosis and gastrointestinal bleeding.^45^ Based on the side effects of corticosteroid therapy, a single application of corticosteroid drugs such as dexamethasone should be discontinued and a combination with other drugs should be used to reduce adverse drug reactions. Whether MSCs combined with glucocorticoids have an improved therapeutic effect on silicosis should be studied. Glucocorticoids are commonly administered systemically or topically; however, atomization inhalation using a combination of a corticosteroid hormone (for example, glucocorticoid drugs such as budesonide) with BMSCs evenly distributes the drug directly in the bronchi and alveoli and reduces the airway mucosal glands, thereby relieving the clinical symptoms. The combination of novel and traditional therapies reduces drugs dose and the side effects of corticosteroids. A clinical study has proved the safety of this new therapy.^46^ However, further studies are required for its application in silicosis.

The application of stem cell therapy is still controversial and limited because of several factors, such as malignant proliferation and tumorigenesis of MSCs,^47^ and major ethical issues. The duration of the effect of MSCs and the infusion time, dose and route still need to be further explored. ‘Cell free therapy’,^48^, ^49^ the genetic modification of stem cell therapy^50^, ^51^ and joint application of drugs have also drawn attention, which provide a better platform and choices for disease treatment, but further laboratory and clinical researches are needed to improve exosome isolation methods, dose selection, and gene modification techniques. This study aimed to provide new evidence for the treatment and research of silicosis.

## 5 CONCLUSION

In this study, the results showed that BMSCs could alleviate silica‐induced pulmonary fibrosis in rats by regulating inflammatory response and EMT process and reducing ECM deposition. Furthermore, silica stimulation activates the TGF‐β/Smad pathway, whereas BMSCs could inhibit this activation. Ultimately, these findings suggest that BMSCs may be a promising therapeutic strategy for treating silicosis.

## CONFLICT OF INTEREST

The authors confirm that there are no conflicts of interest.

## AUTHOR CONTRIBUTION

**Jingjing Wei:** Conceptualization (lead); Resources (equal); Writing‐original draft (lead). **Qiuyan Zhao:** Conceptualization (equal); Software (equal). **Guo Yang:** Methodology (equal). **Ruoxuan Huang:** Investigation (equal); Visualization (equal). **Chao Li:** Formal analysis (equal); Software (equal). **Yuanmeng Qi:** Methodology (equal). **Changfu Hao:** Project administration (equal). **Wu Yao:** Conceptualization (equal); Funding acquisition (equal); Supervision (equal); Validation (equal).

## Supporting information

Supplementary Material

## References

[1] Rimal B , Greenberg AK , Rom WN . Basic pathogenetic mechanisms in silicosis: current understanding. Curr Opinion Pulmon Med. 2005;11(2):169‐173. 10.1097/01.mcp.0000152998.11335.24 15699791

[2] Leso V , Fontana L , Romano R , Gervetti P , Iavicoli I . Artificial stone associated silicosis: a systematic review. Int J Environ Res Public Health. 2019;16(4):568. 10.3390/ijerph16040568 PMC6406954 30781462

[3] Shi P , Xing X , Xi S , et al. Trends in global, regional and national incidence of pneumoconiosis caused by different aetiologies: an analysis from the Global Burden of Disease Study 2017. Occup Environ Med. 2020;77(6):407‐414. 10.1136/oemed-2019-106321 32188634

[4] Leung CC , Yu IT , Chen W . Silicosis. Lancet. 2012;379(9830):2008‐2018. 10.1016/S0140-6736(12)60235-9 22534002

[5] Hoy R , Chambers DC . Silicosis: an ancient disease in need of a dose of modern medicine. Respirology. 2020;25(5):464‐465. 10.1111/resp.13766 31970870

[6] Naji A , Eitoku M , Favier B , Deschaseaux F , Rouas‐Freiss N , Suganuma N . Biological functions of mesenchymal stem cells and clinical implications. Cell Mol Life Sci. 2019;76(17):3323‐3348. 10.1007/s00018-019-03125-1 31055643 PMC11105258

[7] Gorabi AM , Kiaie N , Barreto GE , Read MI , Tafti HA , Sahebkar A . The therapeutic potential of mesenchymal stem cell‐derived exosomes in treatment of neurodegenerative diseases. Mol Neurobiol. 2019;56(12):8157‐8167. 10.1007/s12035-019-01663-0 31197655

[8] Mahdavi Gorabi A , Banach M , Reiner Ž , et al. The role of mesenchymal stem cells in atherosclerosis: prospects for therapy via the modulation of inflammatory milieu. J Clin Med. 2019;8(9):1413. 10.3390/jcm8091413 PMC6780166 31500373

[9] Zhou J , Jiang L , Long X , et al. Bone‐marrow‐derived mesenchymal stem cells inhibit gastric aspiration lung injury and inflammation in rats. J Cell Mol Med. 2016;20(9):1706‐1717. 10.1111/jcmm.12866 27061967 PMC4988291

[10] Savukinas UB , Enes SR , Sjöland AA , Westergren‐Thorsson G . Concise review: the bystander effect: mesenchymal stem cell‐mediated lung repair. Stem Cells. 2016;34(6):1437‐1444. 10.1002/stem.2357 26991735

[11] Huang J , Huang J , Ning X , et al. CT/NIRF dual‐modal imaging tracking and therapeutic efficacy of transplanted mesenchymal stem cells labeled with Au nanoparticles in silica‐induced pulmonary fibrosis. J Materials Chem. 2020;8(8):1713‐1727. 10.1039/c9tb02652e 32022096

[12] Stone RC , Pastar I , Ojeh N , et al. Epithelial‐mesenchymal transition in tissue repair and fibrosis. Cell Tissue Res. 2016;365(3):495‐506. 10.1007/s00441-016-2464-0 27461257 PMC5011038

[13] Diepenbruck M , Christofori G . Epithelial‐mesenchymal transition (EMT) and metastasis: yes, no, maybe? Curr Opin Cell Biol. 2016;43:7‐13. 27371787 10.1016/j.ceb.2016.06.002

[14] Dongre A , Weinberg RA . New insights into the mechanisms of epithelial‐mesenchymal transition and implications for cancer. Nat Rev Mol Cell Bio. 2019;20(2):69‐84. 10.1038/s41580-018-0080-4 30459476

[15] Lamouille S , Xu J , Derynck R . Molecular mechanisms of epithelial‐mesenchymal transition. Nat Rev Mol Cell Biol. 2014;15(3):178‐196. 10.1038/nrm3758 24556840 PMC4240281

[16] Yao Y , Chen R , Wang G , Zhang Y , Liu F . Exosomes derived from mesenchymal stem cells reverse EMT via TGF‐β1/Smad pathway and promote repair of damaged endometrium. Stem Cell Res Ther. 2019;10(1): 10.1186/s13287-019-1332-8 PMC6664513 31358049

[17] Song Y , Peng C , Lv S , et al. Adipose‐derived stem cells ameliorate renal interstitial fibrosis through inhibition of EMT and inflammatory response via TGF‐β1 signaling pathway. Int Immunopharmacol. 2017;44:115‐122. 10.1016/j.intimp.2017.01.008 28092863

[18] Argun Baris S , Vural C , Yaprak B , et al. The effects of sildenafil on smoke induced lung inflammation in rats. Malaysian J Pathol. 2016;38(1):39‐44. 27126663

[19] Hübner RH , Gitter W , El Mokhtari NE , et al. Standardized quantification of pulmonary fibrosis in histological samples. Biotechniques. 2008;44(4):507‐517. 10.2144/000112729 18476815

[20] Chen S , Cui G , Peng C , et al. Transplantation of adipose‐derived mesenchymal stem cells attenuates pulmonary fibrosis of silicosis via anti‐inflammatory and anti‐apoptosis effects in rats. Stem Cell Res Ther. 2018;9(1): 10.1186/s13287-018-0846-9. PMC5909257 29673394

[21] Choi M , Ban T , Rhim T . Therapeutic use of stem cell transplantation for cell replacement or cytoprotective effect of microvesicle released from mesenchymal stem cell. Mol Cells. 2014;37(2):133‐139. 10.14348/molcells.2014.2317 24598998 PMC3935626

[22] Phinney DG , Di Giuseppe M , Njah J , et al. Mesenchymal stem cells use extracellular vesicles to outsource mitophagy and shuttle microRNAs. Nat Commun. 2015;6:8472. 10.1038/ncomms9472 26442449 PMC4598952

[23] Xu C , Zhao J , Li Q , et al. Exosomes derived from three‐dimensional cultured human umbilical cord mesenchymal stem cells ameliorate pulmonary fibrosis in a mouse silicosis model. Stem Cell Res Ther. 2020;11(1):503. 10.1186/s13287-020-02023-9 33239075 PMC7687745

[24] Qi YM , Zhao A , Yang PY , Jin LH , Hao CF . miR‐34a‐5p Attenuates EMT through targeting SMAD4 in silica‐induced pulmonary fibrosis. J Cell Mol Med. 2020;24(20):12219‐12224. 10.1111/jcmm.15853 32929850 PMC7579717

[25] Zhao YL , Hao CF , Bao L , et al. Silica particles disorganize the polarization of pulmonary macrophages in mice. Ecotoxicol Environ Saf. 2020;193:110364. 10.1016/j.ecoenv.2020.110364 32114243

[26] Li X , Wang Y , An G , et al. Bone marrow mesenchymal stem cells attenuate silica‐induced pulmonary fibrosis via paracrine mechanisms. Toxicol Lett. 2017;270:96‐107. 10.1016/j.toxlet.2017.02.016 28232222

[27] Li X , An G , Wang Y , Liang D , Zhu Z , Tian L . Targeted migration of bone marrow mesenchymal stem cells inhibits silica‐induced pulmonary fibrosis in rats. Stem Cell Res Ther. 2018;9(1):335. 10.1186/s13287-018-1083-y 30514375 PMC6280342

[28] King TE , Pardo A , Selman M . Idiopathic pulmonary fibrosis. Lancet. 2011;378(9807):1949‐1961. 10.1016/S0140-6736(11)60052-4 21719092

[29] Wynn TA . Cellular and molecular mechanisms of fibrosis. J Pathol. 2008;214(2):199‐210. 10.1002/path.2277 18161745 PMC2693329

[30] Liu B , Ding F , Hu D , et al. Human umbilical cord mesenchymal stem cell conditioned medium attenuates renal fibrosis by reducing inflammation and epithelial‐to‐mesenchymal transition via the TLR4/NF‐kappaB signaling pathway in vivo and in vitro. Stem Cell Res Ther. 2018;9(1):7. 10.1186/s13287-017-0760-6 29329595 PMC5767037

[31] Wang Z , Li S , Wang Y , Zhang X , Chen L , Sun D . GDNF enhances the anti‐inflammatory effect of human adipose‐derived mesenchymal stem cell‐based therapy in renal interstitial fibrosis. Stem cell research. 2019;41:101605. 10.1016/j.scr.2019.101605 31706095

[32] Xu J , Lamouille S , Derynck R . TGF‐beta‐induced epithelial to mesenchymal transition. Cell Res. 2009;19(2):156‐172. 10.1038/cr.2009.5 19153598 PMC4720263

[33] Lim J , Thiery JP . Epithelial‐mesenchymal transitions: insights from development. Development (Cambridge, England). 2012;139(19):3471‐3486. 10.1242/dev.071209 22949611

[34] Gad ES , Salama AAA , El‐Shafie MF , Arafa HMM , Abdelsalam RM , Khattab M . The anti‐fibrotic and anti‐inflammatory potential of bone marrow‐derived mesenchymal stem cells and nintedanib in bleomycin‐induced lung fibrosis in rats. Inflammation. 2020;43(1):123‐134. 10.1007/s10753-019-01101-2 31646446

[35] Li T , Yan Y , Wang B , et al. Exosomes derived from human umbilical cord mesenchymal stem cells alleviate liver fibrosis. Stem Cells Dev. 2013;22(6):845‐854. 10.1089/scd.2012.0395 23002959 PMC3585469

[36] Zhang L , Zhou D , Li J , et al. Effects of bone marrow‐derived mesenchymal stem cells on hypoxia and the transforming growth Factor beta 1 (TGFβ‐1) and SMADs pathway in a mouse model of cirrhosis. Med Sci Monit. 2019;25:7182‐7190. 10.12659/msm.916428 31550244 PMC6775794

[37] Lv S , Liu G , Sun A , et al. Mesenchymal stem cells ameliorate diabetic glomerular fibrosis in vivo and in vitro by inhibiting TGF‐β signalling via secretion of bone morphogenetic protein 7. Diab Vas Dis Res. 2014;11(4):251‐261. 10.1177/1479164114531300 24845071

[38] Jang YO , Cho MY , Yun CO , et al. Effect of function‐enhanced mesenchymal stem cells infected with decorin‐expressing adenovirus on hepatic fibrosis. Stem Cells Transl Med. 2016;5(9):1247‐1256. 10.5966/sctm.2015-0323 27365486 PMC4996441

[39] Hao Y , Baker D , Ten Dijke P . TGF‐β‐mediated epithelial‐mesenchymal transition and cancer metastasis. Int J Mol Sci. 2019;20(11):2767. 10.3390/ijms20112767 PMC6600375 31195692

[40] Olsen TR , Ng KS , Lock LT , Ahsan T , Rowley JA . Peak MSC‐are we there yet? Front Med. 2018;5:178. 10.3389/fmed.2018.00178 PMC6021509 29977893

[41] Sudlow AW , Carey F , Forder R , Rothwell NJ . The role of lipocortin‐1 in dexamethasone‐induced suppression of PGE2 and TNF alpha release from human peripheral blood mononuclear cells. Br J Pharmacol. 1996;117(7):1449‐1456. 10.1111/j.1476-5381.1996.tb15305.x 8730738 PMC1909467

[42] Bando T , Fujimura M , Shinagawa S , et al. Effect of beclomethasone dipropionate inhalation on eosinophilic bronchitis in patients with silicosis. Arzneimittelforschung. 1997;47(12):1370‐1374. 9450166

[43] Goodman GB , Kaplan PD , Stachura I , Castranova V , Pailes WH , Lapp NL . Acute silicosis responding to corticosteroid therapy. Chest. 1992;101(2):366‐370. 10.1378/chest.101.2.366 1735256

[44] Rabolli V , Lo Re S , Uwambayinema F , Yakoub Y , Lison D , Huaux F . Lung fibrosis induced by crystalline silica particles is uncoupled from lung inflammation in NMRI mice. Toxicol Lett. 2011;203(2):127‐134. 10.1016/j.toxlet.2011.03.009 21414392

[45] Rice JB , White AG , Scarpati LM , Wan G , Nelson WW . Long‐term systemic corticosteroid exposure: a systematic literature review. Clin Ther. 2017;39(11):2216‐2229. 10.1016/j.clinthera.2017.09.011 29055500

[46] Xu LH , Ou RQ , Wu BJ , Wang HY , Fang JP , Tan WP . Corticosteroid in combination with leflunomide and mesenchymal stem cells for treatment of pediatric idiopathic pulmonary hemosiderosis. J Trop Pediatr. 2017;63(5):389‐394. 10.1093/tropej/fmx002 28158572

[47] Hmadcha A , Martin‐Montalvo A , Gauthier BR , Soria B , Capilla‐Gonzalez V . Therapeutic potential of mesenchymal stem cells for cancer therapy. Front Bioeng Biotechnol. 2020;8:43. 10.3389/fbioe.2020.00043 32117924 PMC7013101

[48] Lai RC , Yeo RW , Lim SK . Mesenchymal stem cell exosomes. Semin Cell Dev Biol. 2015;40:82‐88. 10.1016/j.semcdb.2015.03.001 25765629

[49] Keshtkar S , Azarpira N , Ghahremani MH . Mesenchymal stem cell‐derived extracellular vesicles: novel frontiers in regenerative medicine. Stem Cell Res Ther. 2018;9(1):63. 10.1186/s13287-018-0791-7 29523213 PMC5845209

[50] Hu C , Li L . Preconditioning influences mesenchymal stem cell properties in vitro and in vivo. J Cell Mol Med. 2018;22(3):1428‐1442. 10.1111/jcmm.13492 29392844 PMC5824372

[51] Hamada H , Kobune M , Nakamura K , et al. Mesenchymal stem cells (MSC) as therapeutic cytoreagents for gene therapy. Cancer Sci. 2005;96(3):149‐156. 15771617 10.1111/j.1349-7006.2005.00032.x PMC11159137

