# Supplementary material for: Mesenchymal stem cells ameliorate silica‐induced pulmonary fibrosis by inhibition of inflammation and epithelial‐mesenchymal transition
Source: J Cell Mol Med. 2021 Jun 2;25(13):6417–28. doi: 10.1111/jcmm.16621 (PMC8256359; doi:10.1111/jcmm.16621)
Supplement: Supplementary file 1 — Supplementary Material [file JCMM-25-6417-s001.docx]

Supplementary information

Table S1. Primer sequence.

| **Gene name** | **Primer sequence(5’-3’)** | |
| --- | --- | --- |
| Collagen Ⅰ | Forward: | TGTTGGTCCTGCTGGCAAGAATG |
|  | Reverse: | GTCACCTTGTTCGCCTGTCTCAC |
| Fibronectin | Forward: | AGGCACAAGGTCCGAGAAGAGG |
|  | Reverse: | CATGAGTCATCCGTAGGCTGGTTC |
| *Tnfa* | Forward: | ATGGGCTCCCTCTCATCAGTTCC |
|  | Reverse: | GCTCCTCCGCTTGGTGGTTTG |
| *Il6* | Forward: | ACTTCCAGCCAGTTGCCTTCTTG |
|  | Reverse: | TGGTCTGTTGTGGGTGGTATCCTC |
| *Il1b* | Forward: | CTCACAGCAGCATCTCGACAAGAG |
|  | Reverse: | TCCACGGGCAAGACATAGGTAGC |
| *Tgfb1* | Forward: | CCTGGAAAGGGCTCAACAC |
|  | Reverse: | CAGTTCTTCTCTGTGGAGCTG |
| E-cadherin | Forward: | CCTACAATGCTGCCATCGCCTAC |
|  | Reverse: | GGGTAACTCTCTCGGTCCAGTCC |
| Vimentin | Forward: | GTCCGTGTCCTCGTCCTCCTAC |
|  | Reverse: | AGGTGCGGGTGGATGTGGTC |
| GAPDH | Forward: | GGCACAGTCAAGGCTGAGAATG |
|  | Reverse: | ATGGTGGTGAAGACGCCAGTA |


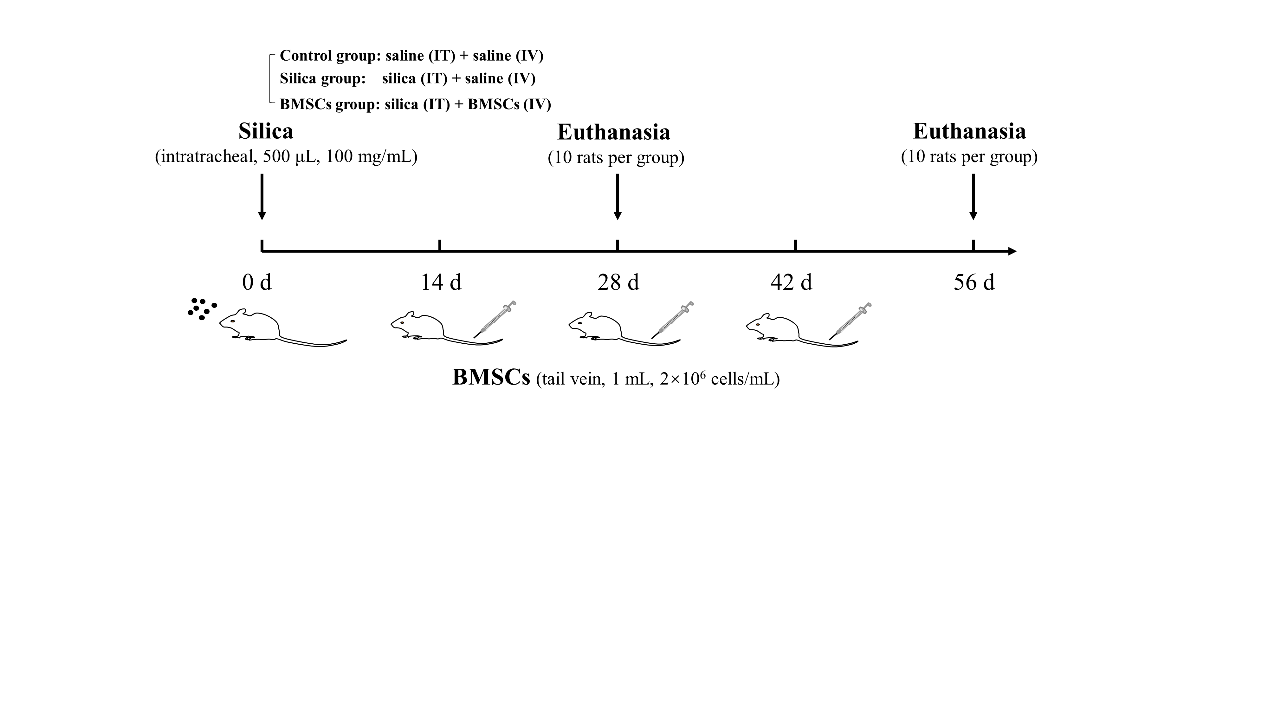


Figure S1. Study design. *IT*-intratracheal，*IV*- intravenous.


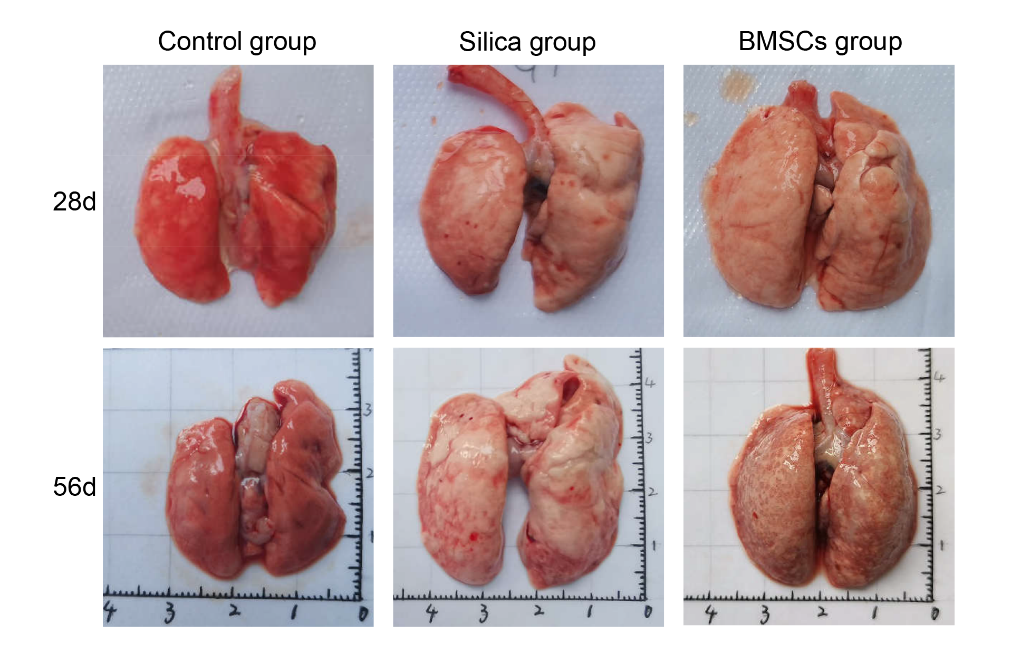


Figure S2. BMSCs improved pulmonary morphology. The pulmonary morphology of each group after rats were exposed to silica for 28 and 56 days.


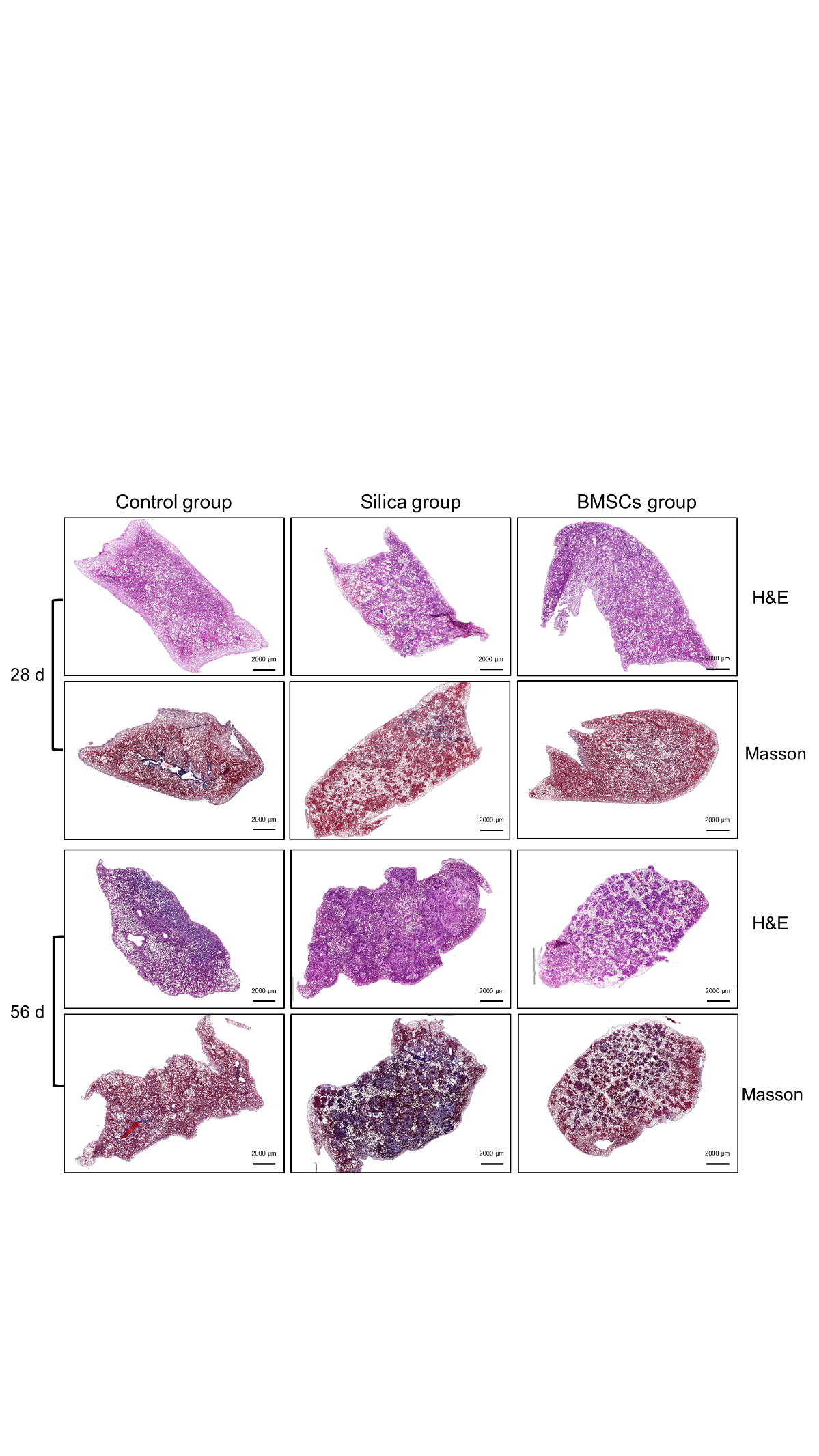


Figure S3. BMSCs improved pathological changes. H&E staining and Masson's trichrome staining were preformed to detect the pathological changes of lung tissues after rats were exposed to silica for 28 and 56 days, respectively (whole section).
